# Invariant Natural Killer T Cells Shape the Gut Microbiota and Regulate Neutrophil Recruitment and Function During Intestinal Inflammation

**DOI:** 10.3389/fimmu.2018.00999

**Published:** 2018-05-07

**Authors:** Sj Shen, Kathryn Prame Kumar, Dragana Stanley, Robert J. Moore, Thi Thu Hao Van, Shu Wen Wen, Michael J. Hickey, Connie H. Y. Wong

**Affiliations:** ^1^Centre for Inflammatory Diseases, Monash University Department of Medicine, Monash Medical Centre, Clayton, VIC, Australia; ^2^School of Health Medical and Applied Sciences, Central Queensland University, Rockhampton, QLD, Australia; ^3^Infection and Immunity Program, Department of Microbiology, Monash Biomedicine Discovery Institute, Monash University, Melbourne, VIC, Australia; ^4^School of Science, RMIT University, Melbourne, VIC, Australia

**Keywords:** neutrophils, colitis, inflammation, mucosal immunology, innate immunity

## Abstract

Invariant natural killer T (iNKT) cells and neutrophils play an increasingly important part in the pathogenesis of inflammatory diseases, but their precise roles in modulating colitis remain unclear. Previous studies have shown important interplays between host immune system and the gut microbiota, and the resulting modulation of inflammation. However, the interactions between iNKT cells, neutrophil and gut microbiota in regulating colitis pathology are poorly understood. Here, we show iNKT cell-deficient *J*α*18^−/−^* mice display reduced dextran sodium sulfate (DSS)-induced colonic inflammation compared to their wild-type (WT) counterparts. We reveal that there is a distinct gut microbiota shaped by the absence of iNKT cells, which comprises of microorganisms that are associated with protection from colonic inflammation. Additionally, the reduced inflammation in *J*α*18^−/−^* mice was correlated with increased expressions of neutrophil chemoattractant (*Cxcl1* and *Cxcl2*) and increased neutrophil recruitment. However, these neutrophils were recruited to the colon at day 3 of our model, prior to observable clinical signs at day 5. Further analysis shows that these neutrophils, primed by the microbiota shaped by the lack of iNKT cells, exhibit anti-inflammatory and immune-modulatory properties. Indeed, depletion of neutrophils in DSS-treated *J*α*18^−/−^* mice demonstrates that neutrophils confer an anti-colitogenic effect in the absence of iNKT cells. Thus, our data supports a changing dogma that neutrophils possess important regulatory roles in inflammation and highlights the complexity of the iNKT cell–microbiota–neutrophil axis in regulating colonic inflammation.

## Introduction

Inflammatory bowel disease describes a group of chronic and idiopathic inflammatory diseases of the gastrointestinal tract and includes Crohn’s disease (CD) and ulcerative colitis (UC). Emerging evidence indicates that a combination of genetic and environmental risk factors predisposes the host to colonic inflammation and immune cell infiltration, with accumulation of neutrophils considered to be a histological hallmark of UC (1). Despite earlier findings showing a pathogenic role for neutrophils (2), there is a growing body of evidence to demonstrate that neutrophils participate in protective functions in colitis (3). In particular, interleukin- (IL-) 22 produced by neutrophils has been shown to reduce epithelial damage and inflammation in the colon, indicating that neutrophils act to promote wound healing and tissue repair (3). It is noteworthy that therapies aimed at inhibiting leukocyte recruitment, such as blockade of P-selectin glycoprotein ligand-1 or very late antigen-4 present on leukocytes have shown promise in attenuating dextran sodium sulfate (DSS)-induced colitis (4). Despite this, strategies to inhibit other adhesion molecules, such as L-selectin, or depletion of neutrophils, have resulted in aggravated pathology in multiple rodent models of colitis (5). Taken together, the findings from these studies indicate that treatment regimens targeting neutrophils and their recruitment to the colon have heterogeneous effects. As such, a better understanding of the mechanisms underlying neutrophil recruitment and their function is required for the development of more effective therapy.

Recent studies demonstrate that invariant natural killer T (iNKT) cells (a subset of NKT cells that has a restricted repertoire of T cell receptors) are key regulators of neutrophil recruitment and function in various tissues and pathological conditions (6–10). iNKT cells are unique immune regulatory cells that produce both pro- and anti-inflammatory cytokines, allowing them to alter leukocyte polarization and responses (7, 10, 11). There is evidence for the interactions between iNKT cells and neutrophils, in that iNKT cell-deficient mice have more neutrophils in the lungs early in *Pseudomonas aeruginosa* infection (8). Clearly, the capacity of iNKT cells to regulate neutrophil recruitment and function makes it a potentially important aspect of colitis pathogenesis.

The host immune response to colitis pathology is further complicated by the contribution of the gut microbiota. There is accumulating evidence to suggest that bidirectional interaction between the immune system and gut microbiota significantly impacts the outcome of several inflammatory diseases (12). As such, there is renewed interest on the potential of altering the gut microbiota as a novel therapeutic method for inflammatory gut conditions (12). Needless to say, colitis has been one of the most studied inflammatory diseases in relation to the gut microbiota (13). A gut microbiota that shapes the host immune response to promote inflammation was found to be detrimental to disease outcome (14). However, whether iNKT cells control the microbiota composition and impact on leukocyte recruitment and function is currently unknown. In this study, we used mice deficient or depleted of iNKT cells in a DSS-induced model of colitis to examine the role of iNKT cells in regulating the recruitment of neutrophils and resultant colitis pathology. We showed that iNKT cells have an active role in altering the gut microbiota and susceptibility to colitis; and that in the absence of iNKT cells, neutrophils take on an anti-inflammatory role in the inflamed colon. The findings from this study indicate that modulating iNKT cell–microbiota–neutrophil interactions may present as a novel therapeutic avenue for colitis.

## Materials and Methods

### Mice

Six- to eight-week-old male C57BL/6J (wild type; WT), *Cxcr6^gfp/+^* (15), *Cd1d^−/−^* (16), *J*α*18^−/−^* (17), and *LysM^eGFP^* (18) mice (all on a C57BL/6J background) were bred and kept under specific pathogen-free conditions at the Monash Medical Centre Animal Facility. Following transportation, mice were acclimatized for a minimum period of 7 days before use. All mice were housed in groups of no more than 5 animals in each cage after weaning, in a 12-h light–dark cycle and a temperature-controlled environment. Water and food pellets (Irradiated Rat and Mouse, Specialty Feeds, Australia) were provided *ad libitum*, and cages were changed weekly, unless otherwise specified. All experimental procedures were performed in accordance with protocols approved by the Monash Medical Centre B Animal Ethics Committee (MMCB/2015/15).

### Mouse Model of Intestinal Inflammation

Adult male mice were provided with 2% (w/v) dextran sodium sulfate (DSS; molecular weight 36–50 kDa, MP Biomedicals) in autoclaved drinking water for a maximum of 7 days, and weighed and monitored daily. Clinical scoring included general symptoms of activity (0: normal, 1: isolated, 2: huddled/inactive, 3: moribund), coat (0: normal, 1: rough, 2: unkempt, 3: severe hair loss/bleeding), and dehydration (0: none, 1: skin less elastic, 2: skin tenting, 3: skin tenting and eyes sunken), and colitis-specific symptoms of percentage weight loss from the previous day (0: none, 1:0–3%, 2:3–6%, 3:6–10%, 4:>10%), stool consistency (0: normal, 1: soft, 2: loose, 3: liquid, 4: diarrhea), and stool blood content (0: none, 1: visible blood in stool). Control mice were provided with autoclaved water without DSS (H_2_O controls). To aid monitoring, cage bedding and water were not changed during the period of DSS induction of colitis.

In experiments where required, blockade of antigen presentation by CD1d was achieved by intraperitoneal (i.p.) injections of 200 µg rat anti-mouse CD1d-blocking antibody (α-CD1d; 1B1, BD Biosciences) or its isotype control antibody (A95-1, BD Biosciences) at day 0; depletion of iNKT cells was achieved through i.p. injection of NKT14 [200 µg, NKT Therapeutics Inc. (19)] at day 0; depletion of neutrophils was achieved by injections of 500 µg anti-mouse Ly6G (αLy6G; 1A8, Bio X Cell) from 1 day prior to the start of DSS administration, and every second day thereafter.

### Colon Morphology and Histology Scoring

At the experimental endpoint, mice were anesthetized with isoflurane, and blood collected using cardiac puncture. A midline incision was made to isolate and excise the colon, and colon length was measured. The distal colon was collected and fixed in 10% formaldehyde for 24 h, paraffin-embedded, cut into 4 µm sections, and stained using hematoxylin and eosin (H&E). Images of the colon sections were taken at 4× magnification using the Leica DM LB widefield microscope and MC120 HD camera (Leica), and scored following blinding and randomization. Parameters for histology scoring includes the level of tissue involvement (0: none, 1: mucosa, 2: mucosa and sub-mucosa, 3: sub-mucosa-transmural, 4: transmural), level of inflammation (0: none, 1: mild, 2: moderate, 3: severe, 4: severe with gastrointestinal-associated lymphoid tissue involvement), involvement of crypt and epithelium (0: none, 1: surface, 2:2/3 basal, 3: crypt and goblet cell loss, 4: crypt and goblet cell destruction with hyperplasia, surface epithelial destruction), and level of lamina propria/sub-mucosa edema (0: none, 1: mild, 2: moderate, 3: moderate-severe, 4: severe). Following collection of the distal colon for histology, the rest of the colon was used for flow cytometric analysis, or sectioned into two parts for myeloperoxidase assay (middle section) and RNA isolation (proximal section).

### Fecal DNA Extraction and 16S rRNA Gene Amplicon Sequencing and Bioinformatics

Fecal samples were collected directly from mice into individually labeled tubes in sterile conditions. The samples were immediately snap frozen in liquid nitrogen, and stored at −80°C. Extraction of fecal DNA was performed using the Isolate II Genomic DNA Kit (Bioline, USA) in accordance with manufacture’s protocol. PCR (30 cycles), using 50 ng of fecal-derived DNA as template, was performed using Q5 DNA polymerase (New England BioLabs) with a primer set selected to amplify the V3–V4 region of the gene encoding the 16S rRNA (forward: ACTCCTACGGGAGGCAGCAG; and reverse: GGACTACHVGGGTWTCTAAT). Equal quantities of each amplicon were pooled, and sequencing was performed on an Illumina MiSeq (2 × 300 bp), following the method detailed by Fadrosh et al. (20). Data analysis was performed using QIIME 1.9.1 software (21), and sequences were joined using the fastq-join method. The maximum allowed percentage difference within the overlapping region was zero. Sequences were de-multiplexed using the QIIME split library protocol, keeping only sequences with a Phred quality score >20. The data set was inspected for chimeric sequences using Pintail (22). Operational taxonomic units (OTUs) were picked using Uclust algorithm (23), and the taxonomy was assigned against the GreeneGene database (24). OTUs with less than 0.01% abundance or those assigned to Cyanobacteria were filtered out. Further data analysis was done using Calypso (25). Other than UniFrac and alpha diversity measures that used rarefied data, all statistical analysis was carried out using OTU table that was log 2 transformed and Cumulative Sum Scaling (CSS) normalized (26).

### Cytometric Bead Array (CBA)

Serum collected at the end of experiments was stored at −80°C until use for CBA. Procedures for CBA were performed as per manufacture’s protocol (BD Biosciences). Samples were incubated with beads specific for IFN-γ, IL-2, IL-4, IL-6, and IL-17, and measured using a Navios flow cytometer (Beckman Coulter). The median fluorescent intensity for each sample was calculated, and quantified relative to a standard curve to obtain the concentration of each cytokine per sample.

### RNA Isolation and qRT-PCR

Total RNA from colon tissue was extracted using TRI-reagent (Sigma) as per the manufacturer’s protocol. Since DSS has been shown to disrupt the activity of reverse transcriptase, the precipitated RNA samples were then purified of DSS through a lithium chloride purification step (27), and treated with DNase (Invitrogen). For flow cytometry-sorted CD45^+^Ly6G^+^ neutrophils (BD FACSARIA III), RNA isolation with DNase treatment was performed using the RNeasy Mini kit (Qiagen), as per the manufacturer’s protocol. The purified RNA was reverse transcribed into cDNA using SuperScript III synthesis system (Invitrogen). Quantitative PCR was performed using Power SYBR Green PCR Master Mix (Applied Biosystems), targeting expression of *18S, Chil3* (Ym-1), *Cxcl1, Cxcl2, Il1b, Il18, Mrc1* (CD206) *Nos2* (iNOS), *Tgfb*, and *Tnfa* (primer sequences can be found at Table S1 in Supplementary Material). All samples were run in triplicate and normalized to *18S*. Expression of each gene was expressed as fold change relative to WT H_2_O control, or to WT DSS for experiments involving the depletion of neutrophils.

### Flow Cytometry of Colon and Spleen Leukocytes

Isolated colons were washed in phosphate buffered saline (PBS) to remove fecal matter, then cut into small pieces and washed in Hank’s balanced salt solution (HBSS, Life Technologies). These samples were incubated in HBSS with 10% fetal calf serum (FCS, Life Technologies) and 5 mM EDTA, then digested in 0.5 mg/mL collagenase D for 90 min, and passed through a 70 µm mesh. The cells were resuspended in 40% isotonic Percoll (Sigma), and layered over 80% isotonic Percoll. Gradients were resolved by centrifugation for 20 min at 1000 *g* with no brake, after which leukocytes were collected from the interface. Total colonic leukocyte numbers were counted by hemocytometer. For the spleen, splenocytes were gently pushed out of a needle-punctured spleen, and resuspended in FACS buffer (10% FCS and 5 mM EDTA in PBS). Red blood cells were lysed, and splenocytes were filtered through a 70 µm mesh.

Cell viability was determined using 7-Aminoactinomycin D, and leukocyte populations enumerated using fluorochrome-conjugated monoclonal antibodies against CD45 (30-F11, eBioscience), CD3 (145-2C11, BD Biosciences), Ly6C (AL-21, BD Biosciences), and Ly6G (1A8, Biolegend). Cells were analyzed on a BD FACSCanto II (BD Biosciences) and data analyzed using FlowJo (v10.0.7, Tree Star Inc.), or sorted using Influx sorter (BD Biosciences) or FACSARIA III (BD Biosciences). Populations were defined as CD45^+^ leukocytes, with further separation into CD3^+^ T cells, Ly6C^hi^Ly6G^−^ monocytes, and Ly6G^+^ neutrophils.

### Liver Intravital Microscopy

To examine the effect of DSS-induced intestinal inflammation on iNKT cell trafficking in the liver, intravital scanning disk confocal microscopy of the intact liver was performed on *Cxcr6^gfp/+^* mice, as previously described (28). Mice were anesthetized by i.p. injection of an anesthetic cocktail consisting of 150 mg/kg ketamine hydrochloride and 10 mg/kg xylazine, and the tail vein was cannulated to administer fluorescently labeled antibodies and/or additional anesthetic as required. Mice were placed in a right lateral position on an adjustable microscope stage. A lateral abdominal incision along the costal margin to the mid-axillary line was made to exteriorize the liver, and all exposed tissues were moistened with saline-soaked gauze to prevent dehydration. As positive control for iNKT cell activation, mice were injected with agonist alpha-galactosylceramide (α-GalCer; 2 μg/mouse i.p.) 4 h prior to imaging. Tissue was imaged using spinning disk confocal microscopy (Perkin Elmer). FITC and PE were excited at 488 and 567 nm respectively; in rapid succession and visualized with a 530/60 and 624/40 nm band pass filter, respectively. Typical exposure time for each excitation wavelength was 300 ms. A 512 × 512 pixel image was acquired every 6 s for 10 min. Andor IQ software (Andor) was used to drive the microscope.

The video files were imported into ImageJ, and adjustments to brightness and contrast were made to individual files to allow the best visualization and measurements of cells. Cells were tracked using the MTrackJ plugin [Biomedical Imaging Group Rotterdam (29)] to find a bright centroid within a 51 × 51 pixel box. The selection box is placed on the cell for every frame that the cell is clearly visible in. The displacement of each cell relative to its initial position was automatically calculated, then averaged. The measurements for all cells in at least two fields of views were averaged to form the data point for one individual mouse.

### Colon Intravital Microscopy

To examine the neutrophil–endothelial cell interactions in the colon during DSS-induced colitis, intravital multiphoton microscopy of the intact colon was performed on anesthetized *LysM^eGFP^* mice, with tail vein cannulation as described. Body temperature was maintained using a heat pad. Mice were placed in a supine position, and the colon exteriorized through a midline incision. All exposed tissues were kept moist with saline-soaked gauze to prevent dehydration. The colon was carefully placed between a custom 3D-printed platform and coverslip setup, which was modified from a previously described setup (30). Vacuum grease was applied in between the 3D platform and the coverslip, allowing the colon to sit within a sealed chamber, enabling continued superfusion with a solution of 10^−2^ M atropine in saline.

At least three post-capillary venules of approximately 35–60 µm in diameter were chosen per mouse. Images and videos were acquired using a multiphoton microscope (Leica SP5), equipped with a 20× water-dipping objective (NA 1.0) and a MaiTai pulsed infrared laser (SpectraPhysics) set to an excitation wavelength of 810 nm. A 512 × 512 pixel image was acquired every 1.5 s for 10 min. Labeling of the vasculature was performed by intravenous administration of 2.5 µg PE-conjugated anti-CD31 (clone 390, eBioscience).

The video files were imported into FIJI (v1.51, NIH) and converted into composite-color images. Adjustments to brightness and contrast were made to individual files to allow optimal visualization and measurements of blood vessels and cells. Multiple measurements of the width of blood vessels were taken and averaged, while the length of the vessel was measured along its center, both using the segmented line tool. The number of adherent, intravascular neutrophils was normalized to the vessel surface area and time of recording. Neutrophils were considered adherent if they interacted with the vessel wall for 30 s or more (31). The number of extravascular neutrophils was normalized to the area of colon that was visible in the field of view. The measurements from all fields of view from one mouse were averaged with the final data representing neutrophil numbers from one mouse.

For experiments assessing effects of atropine on the rate of blood perfusion in the colonic vasculature, FITC-conjugated microbeads were injected intravenously (i.v.), and images were acquired at 330 ms intervals for at least 30 s over three different fields of views. The effect of atropine superfusion was assessed with increasing concentrations of atropine (10^−6^–10^−2^ M) in the same mouse.

### Immunofluorescence

Adult mouse colon was separated into 0.5–1 cm segments and embedded in O.C.T. embedding compound. 8 µm sections were taken using a cryostat, fixed using 4% paraformaldehyde and washed using distilled water and TBST. Blocking solution containing 0.5% Triton X-100 was added for 15 min followed by an incubation with FITC-conjugated anti-CD45 (1:100; eBioscience, San Diego, CA, USA) and PE-conjugated anti-FoxP3 (1:50; eBioscience, San Diego, CA, USA) antibodies for 48 h at 4°C. Samples were mounted with ProLong™ Diamond Antifade Mountant with DAPI (Life technologies) and left to cure for 2 h. Samples were imaged the same day using a Nikon C1 (Upright) confocal microscope equipped with a 40× objective lens and running NIS Elements Software (Nikon. Tokyo, Japan). Images were analyzed using ImageJ 2.0 software. CD45^+^FOXP3^+^ cells were considered as regulatory T cells (Tregs).

### Gut Permeability Assay

At the end of the model of colitis, 500 mg/kg of 4.4 kDa fluorescein-isothiocyanate-labeled dextran (FITC-dextran, Sigma) was orally gavaged into mice. After 4 h, mice were anesthetized by isoflurane, and cardiac puncture was performed to collect blood for serum. Samples were stored at −80°C until use. Serum fluorescence was read at gain 75, excitation 485 nm, and emission 520 nm using an Infinite M1000 Pro (Tecan) plate reader and Magellan 7.2 (Tecan) software. Sample concentrations of FITC-dextran translocation were determined relative to that of a standard curve using Prism (GraphPad) software.

### Statistical Analysis

Student’s *t*-test was used to compare the mean between two groups, except for gene expression data, where Mann–Whitney’s *U*-test was performed. Two-way ANOVA with matched time points was used to compare the differences between two groups over time. All data are represented as mean ± SEM, or with a line at the median for gene expression data. Comparisons were considered significant if the *p*-value is <0.05.

## Results

### Mice Deficient of iNKT Cells Are Protected From DSS-Induced Colitis

**Figure 1:**
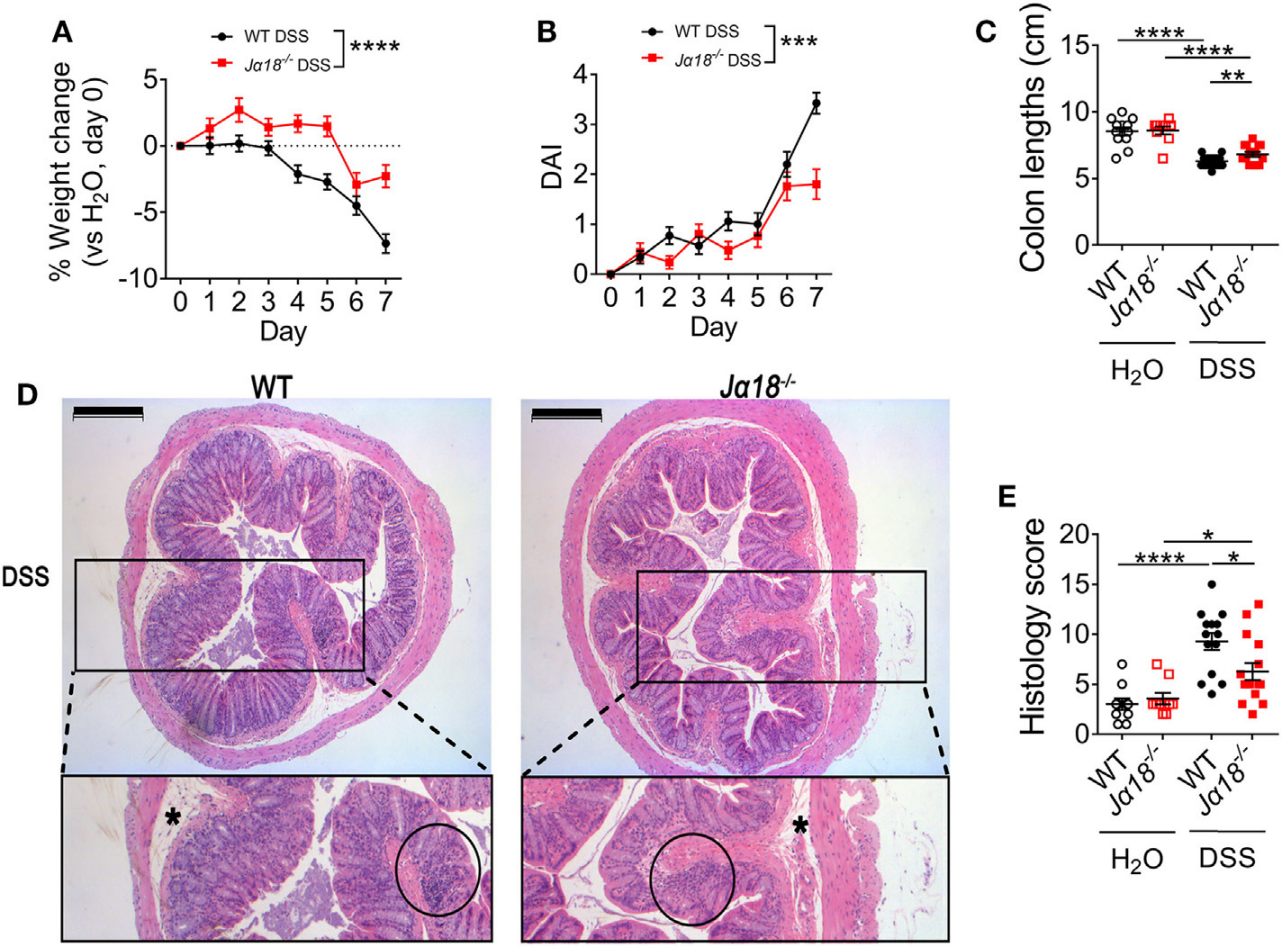
Clinical and histological pathology of 2% dextran sodium sulfate (DSS)-induced colitis in wild type (WT) and *J*α*18*^−/−^ mice. **(A)** Changes in weight of mice treated with DSS were normalized to day 0, then to the respective H_2_O controls. **(B)** Disease activity index (DAI) shown for mice treated with DSS. H_2_O controls showed no visible symptoms. **(C)** At day 7, mice were culled, and the colon lengths measured. **(D)** The distal colon was collected, fixed, and embedded in paraffin. Sections of 4 µm were stained using hematoxylin and eosin. **(E)** Images of the colons were de-identified and randomized. Pathology was scored using four parameters and then totaled. Scale bar represents 200 µm. Asterisk (*) identifies edema. Cellular infiltration is circled. **(A,B)** WT DSS *n* = 35, *J*α*18*^−/−^ DSS *n* = 25. **(C,E)** *n* ≥ 9. All data represent mean ± SEM. Two-way ANOVA with matched time points was used to compare the differences between two groups over time. Student’s *t*-test was performed. Significance is represented by *p < 0.05, **p < 0.01, ***p < 0.001, and ****p < 0.0001. These data were obtained from at least two independent experiments.

To investigate the role of iNKT cells in colitis, DSS colitis was induced in wild type (WT) and iNKT cell-deficient mice (*J*α*18^−/−^* mice). Both WT and *J*α*18^−/−^* mice on normal water had natural weight gain and developed no clinical symptoms, as scored by the DSS-associated disease activity index (DAI) throughout the 7 days of the experimental period. However, administration of DSS caused progressive weight loss in WT mice starting from day 3, when normalized to the weight of H_2_O controls (Figure 1A, non-normalized data in Figure S1A in Supplementary Material). Additionally, DSS-treated WT mice also showed observable clinical symptoms as measured by the DAI such as loose blood-containing stools (Figure 1B) and shortening of the colon length (a surrogate marker of inflammation; Figure 1C). In contrast, *J*α*18^−/−^* mice treated with the same concentration of DSS demonstrated reduced and delayed onset of weight loss (Figure 1A), as well as lower DAI (Figure 1B) and significantly less colon shortening compared to their WT counterparts at the end of the model (Figure 1C).

Blinded histological assessment of colons revealed no differences between WT and *J*α*18^−/−^* mice in the control H_2_O group. Following DSS treatment, both WT and *J*α*18*^−/−^ mice showed increased inflammation (Figures 1D,E). Consistent with the DAI data, colons from *J*α*18*^−/−^ mice exhibited significantly lower histology scores compared to WT mice (Figure 1E). Intriguingly, these DSS-induced differences in pathology between WT and *J*α*18*^−/−^ mice were independent of increased intestinal permeability, which was similar in WT and *J*α*18*^−/−^ mice following DSS treatment (Figure S1B in Supplementary Material). Taken together, these results demonstrate that *J*α*18*^−/−^ mice have less severe colon inflammation following DSS-induced colitis, and suggest that iNKT cells play a key role in contributing to the colonic inflammation and pathology in this model.

### iNKT Cells Shape a Pro-Colitogenic Microbiota

**Figure 2:**
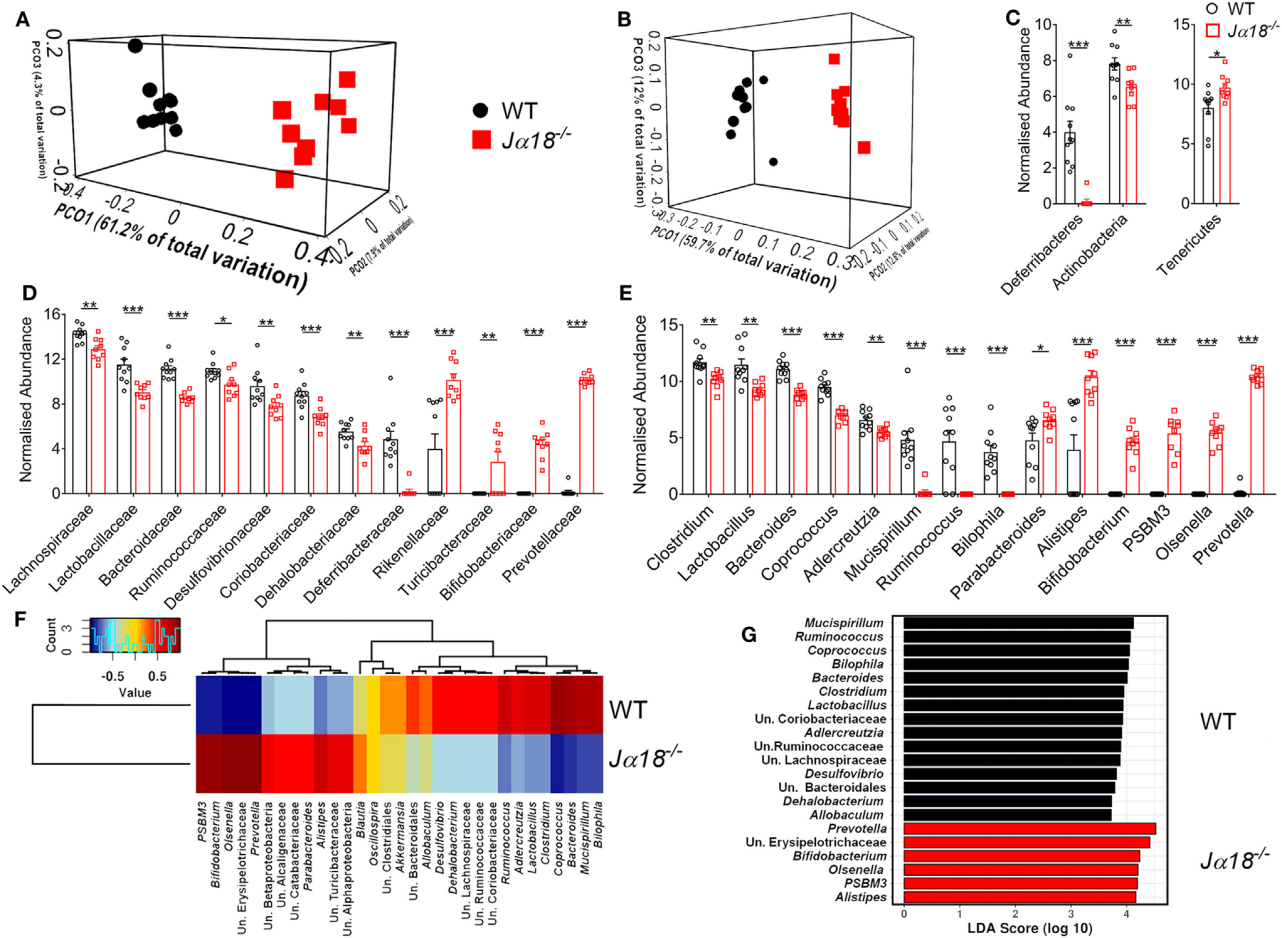
The effect of microbiota differences between wild type (WT) (*n* = 10) and *J*α*18*^−/−^ (*n* = 9) mice. Both **(A)** unweighted and **(B)** weighted UniFrac analysis distance shown as Principal Coordinates Analysis plot, demonstrate strong and significant (both ADONIS *p* < 0.001) difference between the WT and *J*α*18*^−/−^ mice communities. The noticeable differences [*t* test on Cumulative Sum Scaling (CSS) log 2 transformed, CSS normalized data, and FDR corrected *p* < 0.05] were evident at all taxonomic levels, including a **(C)** phylum, **(D)** family, and **(E)** genus. **(F)** Pearson-based genus correlation heatmap and **(G)** LEfSe analysis (black bar: WT, red bar: *J*α*18*^−/−^ mice) show distinctive genus preference by each WT and *J*α*18*^−/−^ mice microbial communities. Significance is represented by *p < 0.05, **p < 0.01, ***p < 0.001. These data were obtained from one experiment (two independent cages/group).

A recent study showed that mice deficit of all types of NKT cells (*Cd1d^−/−^* mice) possess a pro-colitogenic gut microbiota, and the disruption of gut epithelial integrity by DSS exposes the host to the luminal contents, inducing a pro-inflammatory response (32). This raises the possibility that mice with a selective deficiency in iNKT cells may harbor a different microbiota to WT mice, influencing the development of disease. To investigate this, we compared the fecal microbiota composition of naïve WT and *J*α*18^−/−^* mice. We found there were no differences in the richness of microbial communities in the fecal content between WT and *J*α*18^−/−^* mice as measured by α-diversity (Figure S2 in Supplementary Material), but there was a significant difference in the microbial communities between these two strains of mice based on unweighted (different membership; *p* = 6.6 × 10^−4^, Figure 2A) and weighted (different abundance, *p* = 6 × 10^−4^, Figure 2B) UniFrac and Adonis statistics.

Comparison of the fecal microbiota at the phylum level in WT and *J*α*18^−/−^* mice showed significantly elevated relative abundance of Tenericutes in *J*α*18^−/−^* mice, along with a marked reduction of Deferribacteres and Actinobacteria in these mice (Figure 2C). Moreover, at the family level, there was an increased abundance of Rikenellaceae, Turicibacteraceae, Bifidobacteriaceae, and Prevotellaceae (Figure 2D); and a markedly reduced frequency of OTUs belonging to the families of Lachnospiraceae, Lactobacillaceae, Bacteroidaceae, Ruminococcaceae, Desulfovibrionaceae, Coriobacteriaceae, Dehalobacteriaceae, and Defferibacteriaceae in the fecal microbiota of *J*α*18^−/−^* mice (Figure 2D). The genera that were found to be significantly more abundant in *J*α*18^−/−^* microbiota include *Bifidobacterium, PSBM3, Olsenella*, and *Prevotella* (Figure 2E). Intriguingly, we clearly observed a distinction of microbial composition between the strains at the level of genera (Figure 2E) and OTU (species; Figure 2F). Furthermore, differential phylotypes were determined using *t*-test on normalized data with FDR corrected *p* values <0.05 considered significant. At the OTU level (often considered as a species level; clustered at 97% similarity), there were 411 (out of 686) OTUs significantly different (FDR *p* < 0.05) between the fecal microbiota of WT and *J*α*18^−/−^* mice at *p* < 0.01 and a total of 513 OTUs with FDR corrected *p* < 0.05 (see MG-RAST library mgl621715). In WT mice, the fecal microbiota consisted of bacterial species belonging to the genera *Mucispirillum, Ruminococcus, Bacteroides, Clostridium*, and *Lactobacillus*, and these have previously been positively associated with inflammation and colitis (33–35) (Figure 2G). In contrast, the LEfSe analysis demonstrated the major drivers of difference in the microbiota shaped by the absence of iNKT cells were microorganisms previously associated with anti-inflammatory properties, including *Bifidobacterium, PSBM3, Alistipes, Olsenella*, and *Prevotella* (Figure 2G). Taken together, these findings indicate the composition of the colonic microbial communities is significantly affected by the absence of iNKT cells.

### Local Colonic Inflammation Induced by DSS

**Figure 3:**
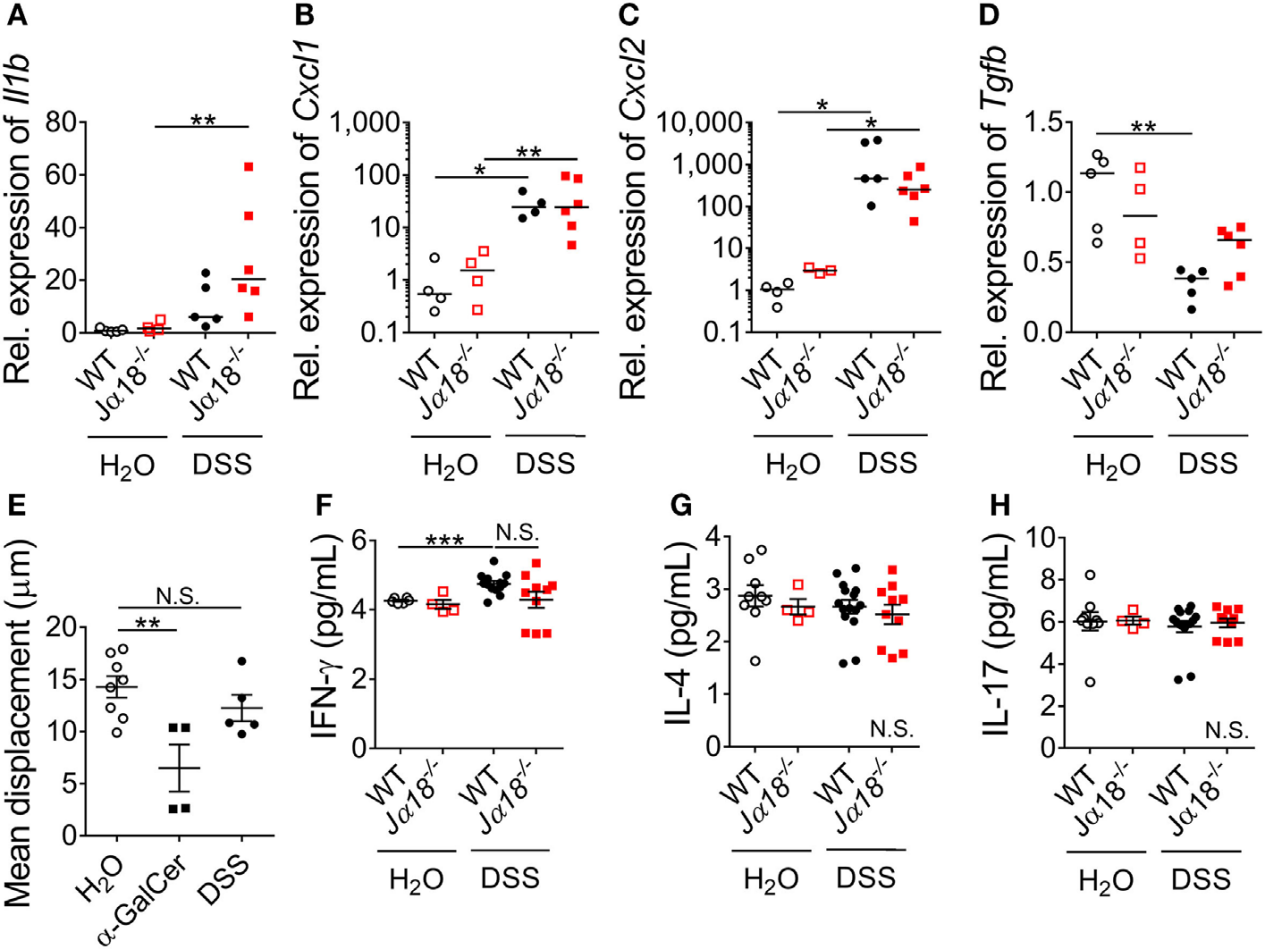
Systemic and local effects of dextran sodium sulfate (DSS) administration in wild type (WT) and *J*α*18*^−/−^ mice. **(A–D)** Expressions of genes in the colon were quantified by qPCR and expressed relative to WT H_2_O controls. Lines represent median and Mann–Whitney *U*-test was performed. **(E)** Mean displacement of liver iNKT cells were quantified in *Cxcr6^gfp/+^* mice provided with normal H_2_O, injected with α-GalCer i.p., or administered 2% DSS for 7 days. **(F–H)** In a separate group, serum cytokine levels of WT and *J*α*18*^−/−^ mice at day 7 were measured using cytometric bead array (CBA). For **(E–H)**, data represent mean ± SEM, and Student’s *t*-test was performed. *n* ≥ 3. Significance is represented by *p < 0.05, **p < 0.01, and ***p < 0.001. N.S. denotes not significant. These data were obtained from at least two independent experiments.

Next, we investigated whether iNKT cells and the gut microbiota shaped by it have a role in regulating colonic inflammation following DSS-induced colitis. Leukocyte recruitment, in particular that of neutrophils, is a histological hallmark of disease (1). Therefore, we examined the gene expression of pro-inflammatory cytokines and chemokines that support the recruitment of leukocytes in the colons of WT and *J*α*18^−/−^* mice. There were no significant differences in the expression of *Il1b, Cxcl1*, and *Cxcl2* between WT and *J*α*18^−/−^* mice in the H_2_O control groups (Figures 3A–C). Following administration of DSS, there were significant increase in the expressions of *Cxcl1* and *Cxcl2* in WT mice following DSS treatment (Figures 3B,C). In *J*α*18^−/−^* mice, the expressions of *Il1b, Cxcl1*, and *Cxcl2* were also significantly higher after DSS treatment (Figures 3A–C). We also examined the gene expression of the anti-inflammatory cytokine, *Tgfb*, and found a DSS-induced reduction in WT mice was not observed in *J*α*18^−/−^* mice (Figure 3D). There were no changes in the expression of *Tnfa* and *Il18* between WT and *J*α*18^−/−^* mice (Figure S3 in Supplementary Material).

In extension to the local changes, we examined whether the absence of iNKT cells and the accompanying alterations in microbiota composition were associated with systemic inflammatory response following DSS-induced colitis. Although iNKT cells have previously been shown to be present at low levels in the colon (36), the liver contains a high abundance of iNKT cells at baseline (37), and is the site where luminal content derived from the gut must undergo filtration before entry into the systemic circulation (38). Therefore, it is feasible that the iNKT cell-dependent effect on inflammation we observed in the colon was dependent on hepatic iNKT cells. To assess whether DSS induction activated hepatic iNKT cells, iNKT cell-reporter (*Cxcr6^gfp/+^*) mice were administered with DSS and liver intravital microscopy was performed. At baseline, hepatic iNKT cells had a mean displacement of 15 µm within the liver sinusoids over 10 min (Figure 3E). Consistent with published reports, the administration of a potent iNKT cell agonist, α-GalCer, resulted in a significant reduction in displacement 4 h after administration (Figure 3E). In contrast, we found no such reduction in the migratory displacement in mice following 7 days of DSS treatment (Figure 3E; Figure S4 in Supplementary Material), suggesting that hepatic iNKT cells were not activated or affected following DSS-induced colitis.

Similarly, when we examined the serum level of cytokines that iNKT cells are known to produce (IFN-γ, IL-4, and IL-17), we observed no changes between baseline and post-DSS nor between WT and *J*α*18^−/−^* mice, except for a significant increase of IFN-γ in WT mice after DSS treatment (Figures 3F–H). In addition, there were no differences in the systemic levels of IL-2 and IL-6 in DSS-treated WT and *J*α*18^−/−^* mice (data not shown). Taken together, these findings indicate that the local colonic milieu in WT and *J*α*18^−/−^* mice, rather than systemic inflammation, supports the recruitment of leukocyte following DSS-induced colitis.

### DSS-Induced Neutrophil Infiltration Is Elevated in Jα18^*−/−*^ Mice

**Figure 4:**
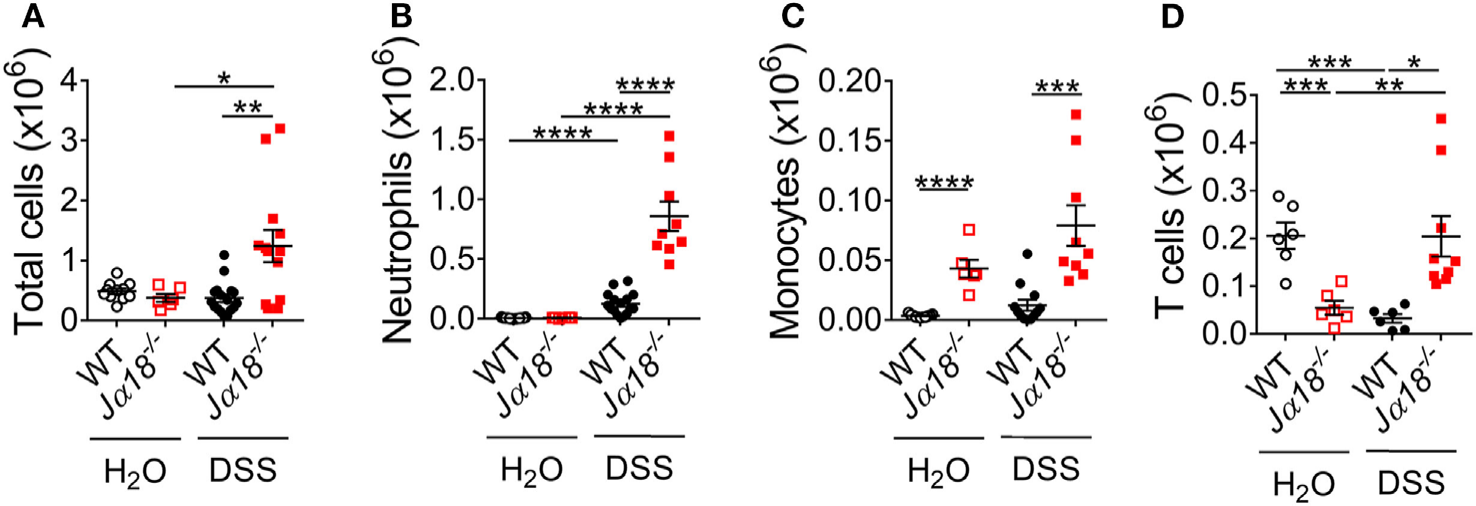
Changes in leukocyte populations following dextran sodium sulfate (DSS)-induced colitis in wild type (WT) and *J*α*18*^−/−^ mice. **(A)** At day 7, total leukocyte numbers from colon of WT and *J*α*18*^−/−^ mice were counted. **(B–D)** Cells were stained with fluorochrome-conjugated antibodies against CD45, CD3, Ly6C, and Ly6G. The number of **(B)** Ly6G^+^ neutrophils, **(C)** Ly6C^hi^Ly6G^−^ monocytes, and **(D)** CD3^+^ T cells were analyzed by flow cytometry. *n* ≥ 6. Data represent mean ± SEM. Student’s *t*-test was performed. Significance is represented by *p < 0.05, **p < 0.01, ***p < 0.001, and ****p < 0.0001. These data were obtained from at least two independent experiments.

To compare the colonic leukocyte infiltrate in WT and *J*α*18^−/−^* mice following DSS-induced colitis, we isolated leukocytes from the colon lamina propria and quantitated total leukocytes, neutrophils, monocytes, and T cells using flow cytometry (Figure S5 in Supplementary Material). At baseline, there were similar numbers of total immune cells in the colons of WT and *J*α*18^−/−^* mice (Figure 4A). However, in the presence of DSS-induced colitis, *J*α*18^−/−^* mice had increased gut immune cell numbers compared to WT DSS animals (Figure 4A). More specific immune cell analysis demonstrated that this increase was due to the greater number of neutrophils (Ly6G^+^; Figure S6A in Supplementary Material; Figure 4B), monocytes (Ly6C^hi^/Ly6G^−^; Figure 4C), and T cells (CD3^+^; Figure 4D). Also evident when assessing the overall immune make-up of CD45^+^ cells was that although DSS-induced colitis significantly increased the proportion of neutrophils in the gut of *J*α*18^−/−^* mice to a greater extent than in WT mice (Figure S6A in Supplementary Material), there were no significant differences between the proportions of monocytes and T cells between post-DSS WT mice and *J*α*18^−/−^* mice (Figures S6B,C in Supplementary Material). These findings suggest iNKT cells play a key role in dampening neutrophil recruitment during colonic inflammation.

To provide further support for the role of iNKT cells in this response, we also administered 2% DSS to *Cd1d^−/−^* mice, which lack all types of NKT cells. Similar to the *J*α*18^−/−^* mice, total leukocyte infiltration was increased in *Cd1d^−/−^* mice compared to WT mice following DSS induction (Figure S6D in Supplementary Material). The proportion of neutrophils in both WT and *Cd1d^−/−^* mice following DSS induction was also increased when compared to H_2_O controls (Figure S6E in Supplementary Material). Furthermore, post-DSS *Cd1d^−/−^* mice also exhibited elevation of neutrophil recruitment to the colon at day 7 of the disease compared to WT mice (*p* = 0.05 Figure S6F in Supplementary Material). These results clearly demonstrate that iNKT cells, rather than other types of NKT cells, play an important role in the regulation of neutrophil recruitment at day 7 of DSS-induced colitis.

### iNKT Cells Induce Accelerated Neutrophil Recruitment Irrespective of Microbial Composition

Our findings so far show that DSS-induced colitis promotes neutrophil recruitment in both WT and *J*α*18^−/−^* mice. However, whether the enhanced neutrophil recruitment in *J*α*18^−/−^* mice is contributed exclusively by the absence of iNKT cells, or in combination with the altered composition of the gut microbiota remains unclear. To address this issue, we examined the effect of iNKT cell-depletion on neutrophil recruitment in a single mouse strain consisting of the same microbiota. To do this, we utilized NKT14, an antibody specific for the invariant chain of the T cell receptor, which has been shown previously to deplete iNKT cells in mice (19). To validate the efficacy of NKT14 for iNKT cell depletion, we injected WT mice with NKT14 at day 0, then used flow cytometry to analyze leukocyte populations in the spleen and blood at days 3, 5, and 7. A single dose of NKT14 was sufficient to induce a sustained reduction in iNKT cells over 7 days in both spleen and the circulation (representative plots in Figure S7 in Supplementary Material, data in Figures S8A,B in Supplementary Material), without altering T cell proportions or numbers (Figures S8C–F in Supplementary Material).

**Figure 5:**
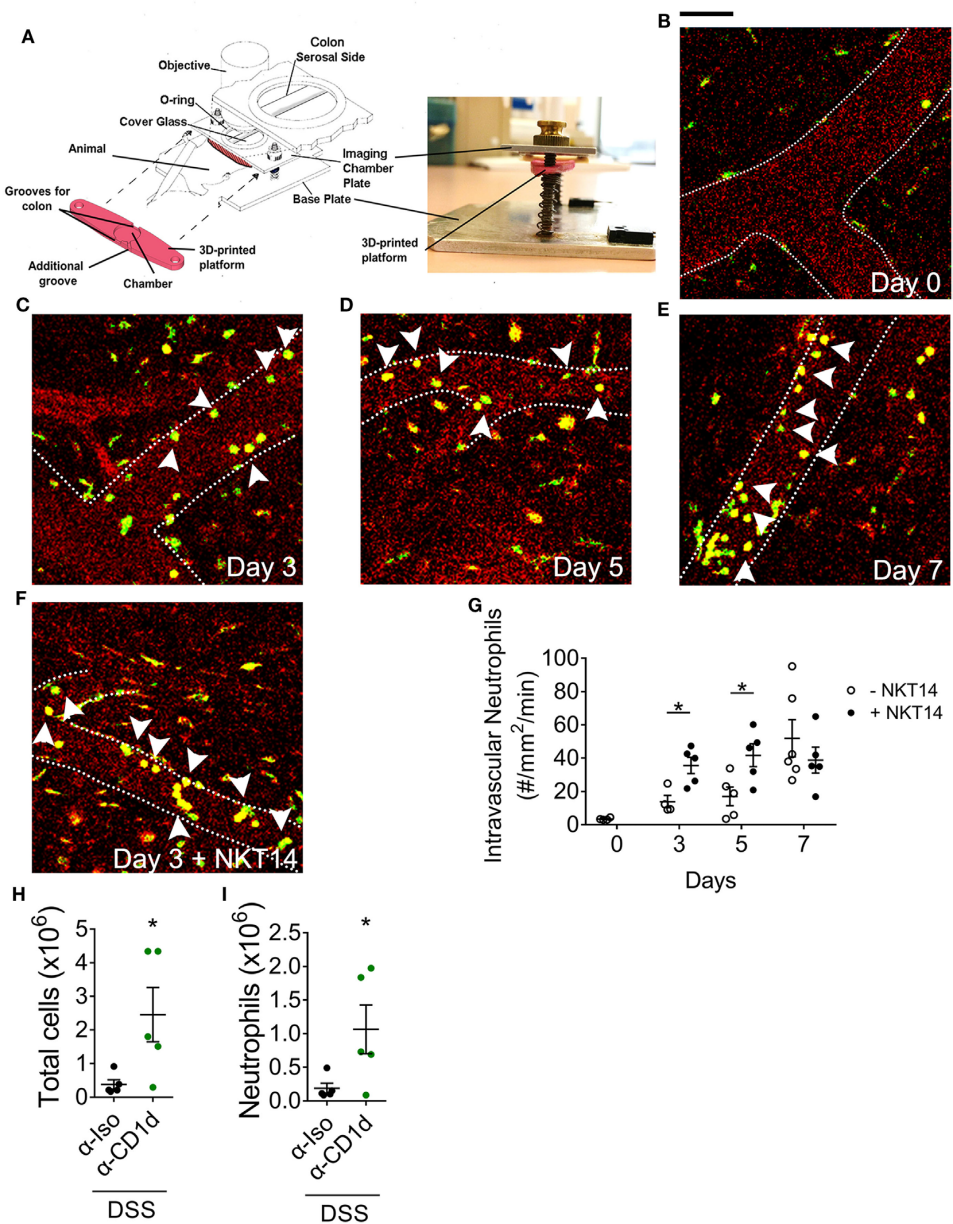
The effect of invariant natural killer T cells on the temporal profile of neutrophils following dextran sodium sulfate (DSS)-induced colitis. **(A)** Setup used to perform intravital microscopy on anesthetized *LysM^eGFP^* mice at days **(B)** 0, **(C)** 3, **(D)** 5, and **(E)** 7 of 2% DSS model. **(F)** NKT14 was injected i.p. at day 0, and microscopy performed at days 3, 5, and 7. **(G)** The number of adherent intravascular neutrophils was enumerated. **(H)** The total numbers of colonic leukocytes and **(I)** neutrophils were analyzed in WT mice administered CD1d-blocking antibody or isotype control at day 7. Scale bar represents 50 µm. Dotted lines represent blood vessel walls. Arrowheads point to intravascular neutrophils. *n* ≥ 4. Data represent mean ± SEM. Student’s *t*-test was performed. Significance is represented by *p < 0.05. CD1d-blocking experiments were performed once. Imaging data were obtained from at least three independent experiments.

To elucidate the effect of iNKT cell-depletion on neutrophil infiltration, we injected neutrophil reporter (*LysM^eGFP^*) mice with NKT14 at day 0 of the DSS model and examined the effect at baseline (day 0), day 3, 5, or 7 using colon intravital microscopy (Figure 5A; Videos S1 and S2 in Supplementary Material). For these experiments we used and validated a new approach for colon intravital microscopy of the colonic microvasculature modified from published methodology (30), avoiding the use of tissue glue, which enables the colon to be in a condition that is much more physiological (Figure S9A in Supplementary Material). In *LysM^eGFP^* mice, circulating neutrophils are detectable as round cells brightly fluorescent *via* GFP (Figures 5B–F). This allowed the differentiation between, and evaluation of, adherent intravascular and transmigrated extravascular neutrophils. At baseline, there were minimal intravascular or extravascular neutrophils in the colon (Figures 5B,G; Figure S9B in Supplementary Material). The number of intravascular neutrophils increased with the progression of DSS-induced colitis (Figures 5C–E,G). Interestingly, depletion of iNKT cells resulted in significantly higher numbers of intravascular neutrophils at 3 and 5 days after DSS induction (Figures 5F,G). The number of extravascular neutrophils also increased with the progression of DSS-induced colitis, however, there were no significant differences following depletion of iNKT cells (Figure S9B in Supplementary Material). These experiments reveal that iNKT cells have the capacity to regulate neutrophil recruitment, regardless of the composition of the microbiota, and particularly in the earlier stages of DSS-induced colitis development.

To examine the mechanisms by which iNKT cells regulate neutrophil recruitment, we examined the role of antigen presentation *via* CD1d (39). Antibody-mediated inhibition of CD1d-dependent antigen presentation resulted in a significant increase in total leukocyte numbers in the colon at day 7 of DSS-induced colitis (Figure 5H). Similar to our findings in *J*α*18^−/−^* mice, there was a significant increase in neutrophil numbers in the colon of post-DSS mice treated with α-CD1d antibody (Figure 5I), though no differences were seen in the proportion of recruited neutrophils (Figure S9C in Supplementary Material). Therefore, by removing the potential effect of different microbiota composition between WT and *J*α*18^−/−^* mice, our findings demonstrate that iNKT cells function to suppress the recruitment of neutrophils in a CD1d-dependent manner following DSS-induced colitis.

### Neutrophil Phenotypes Dictate Outcome of Colonic Inflammation

**Figure 6:**
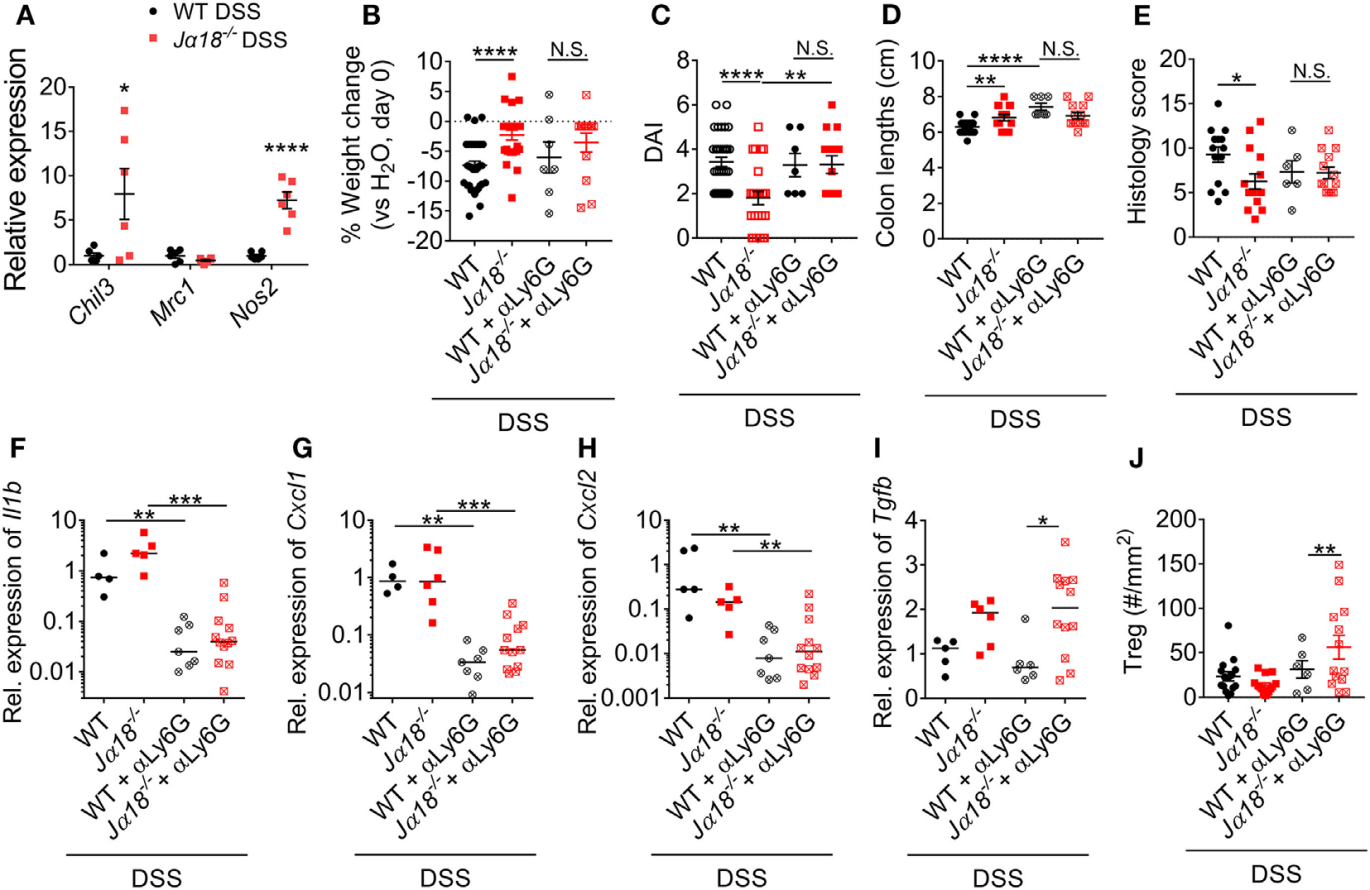
Neutrophil phenotype and function in wild type (WT) and *J*α*18^−/−^* mice. **(A)** Gene expression of isolated neutrophils from the colon of 2% dextran sodium sulfate (DSS)-treated WT and *J*α*18^−/−^* mice was analyzed by qPCR and normalized to that of WT DSS mice. **(B)** Weight change, **(C)** disease activity index (DAI), **(D)** colon lengths, and **(E)** histology scoring of WT and *J*α*18^−/−^* mice with or without depletion of neutrophils were assessed at day 7 of our DSS model. **(F–I)** Colon from these mice was collected, and gene expression analyzed and normalized to WT DSS. **(J)** The number of Tregs in the colon was enumerated by immunofluorescence. *n* ≥ 4. For **(A–E,J)**, data represent mean ± SEM, and Student’s *t*-test was performed. For **(F–I)**, lines represent median, and Mann–Whitney *U*-test was performed. Significance is represented by *p < 0.05, **p < 0.01, ***p < 0.001, and ****p < 0.0001. N.S. denotes not significant. WT and *J*α*18^−/−^* mice DSS data were previously presented in Figure 3, normalized to WT H_2_O. Data of neutrophil depletion were obtained from two independent experiments.

Neutrophils have the capacity to adopt different phenotypes including non-traditional anti-inflammatory functions that may be important in reducing intestinal inflammation (40, 41). However, whether the phenotypes and functions of neutrophils in WT and *J*α*18^−/−^* mice are different is currently unknown. To investigate this, neutrophils were isolated from the colons of WT and *J*α*18^−/−^* mice at day 7 of DSS-induced colitis, and the expression of anti-inflammatory markers were assessed. Neutrophils that infiltrated the colons of *J*α*18^−/−^* mice demonstrated increased expression of *Chil3* (Ym-1) and *Nos2* (iNOS) (Figure 6A), indicating a shift to an anti-inflammatory phenotype (42, 43). To test whether this difference in neutrophil phenotype is important in decreasing intestinal inflammation and confer disease protection, mice were removed of neutrophils using neutrophil depleting antibody and treated with DSS (Figure S10 in Supplementary Material). We have shown that post-DSS *J*α*18^−/−^* mice had reduced weight loss, significantly lower DAI, longer colons, and lower histology score when compared to post-DSS WT mice (Figures 1 and 6B–E). However, after depletion of neutrophils (comparing WT + αLy6G vs. Jα18KO + αLy6G), the difference between WT and Jα18KO is abrogated (Figures 6B–E).

Next, we examined local changes in gene expression in the colon. Depletion of neutrophils resulted in a significant reduction in the expressions of colonic *Il1b, Cxcl1*, and *Cxcl2* irrespective of strain (Figures 6F–H). On the other hand, expression of *Tgfb* was significantly higher in αLy6G-treated *J*α*18^−/−^* mice than in WT mice (Figure 6I). TGF-β has been shown to induce the differentiation of Tregs (44), thus we used immunofluorescence to assess changes in CD45^+^Foxp3^+^ Tregs in the colon. Intriguingly, only in mice without neutrophils and iNKT cells did we observe a significant increase in colonic Tregs (Figure 6J). Taken together, these results indicate that neutrophils recruited to the inflamed colon in the absence of iNKT cells exhibit an anti-inflammatory phenotype. Therefore, in the absence of iNKT cells, neutrophils have important roles in immune regulation that ultimately confers protection to DSS-induced intestinal inflammation.

## Discussion

The exact role of iNKT cells and neutrophils during intestinal inflammation in colitis has been elusive. For example, iNKT cells activated by the treatment of α-GalCer or OCH9 (Th2-polarizing agonist) attenuate the development of DSS-induced colitis (45–47) while in a Th2-predominant model of oxazolone-induced colitis, NKT cells play a crucial role in disease development (48). The findings of this study support a pathogenic role for iNKT cells in DSS-induced colitis. Mice deficient in iNKT cells demonstrated reduced weight loss and colon inflammation following DSS treatment. In addition, these mice harbor an inherently different and anti-inflammatory microbiota. iNKT cells also have important regulatory role in the recruitment and function of neutrophils that is irrespective of the composition of the gut microbiota. An intriguing observation that emerges from this work is that neutrophils recruited to the inflamed colon of *J*α*18^−/−^* mice display evidence of an anti-inflammatory phenotype, and mediate anti-inflammatory effects in DSS-induced colitis. Together, our findings demonstrate that the interactions between iNKT cells, neutrophil, and gut microbiota create a microenvironment that impacts significantly on colonic inflammation following DSS-induced colitis.

One underlying factor that regulates the development of colonic inflammation is the gut microbiota. The microbiota has been shown to be important in the homeostasis of the gut, by modulating (i) the development of gut-associated lymphoid tissue; (ii) the functions of gut immune cells; and (iii) the production of antimicrobial peptides and other immune modulators (12). This complex relationship between host immune cells and microbiota is reiterated by our findings that reveal the absence of iNKT cells which significantly alter the composition of the colonic microbiota. This difference in microbiota is a potential contributor to the lowered disease observed in DSS-treated *J*α*18^−/−^* mice. This hypothesis is supported by a recent study which reported that a gut microbiota harboring a greater proportion of mucin-degrading *Mucispirillum, Bacteroides*, and *Lactobacillus* was pro-colitogenic, rendering animals more susceptible to DSS-induced colitis (32). Similarly, in this study, the colonic microbiota of WT mice, which was affected by greater colonic inflammation compared to *J*α*18^−/−^* mice, is composed of these strains of bacteria. On the contrary, the colonic microbiota of the *J*α*18^−/−^* mice is comprised in high abundance of anti-colitogenic bacteria, including *PSBM3, Prevotella*, and *Bifidobacterium*. Given the complexity of the gut microbiota and their intricate interactions with the host immune system, colonic inflammation and the pathogenesis of colitis are most probably influenced by a group of bacteria, and not individual strains. As such, it is likely that the different overall composition of bacterial strains in the gut of mice lacking iNKT cells skews toward an anti-inflammatory phenotype, which consequently impacts on the associated immune response. Hence, although a previous study reported *Cd1d^−/−^* mice lacking all NKT cells harbor a pro-colitogenic gut microbiota (32), which is in stark contrast to our results in *J*α*18^−/−^* mice, the study also showed that transfer of the pro-colitogenic gut microbiota into WT mice increased their susceptibility to colitis (32). Therefore, it is clear from this and other studies that the composition of the gut microbiota significantly influences disease outcome irrespective of the mouse strain (49), suggesting that different housing environments could underpin the discrepancies between the result of our study and others (32, 45).

Dextran sodium sulfate damages the colonic mucus layer and epithelial barrier, allowing bacterial penetration of mucus layer within 12 h (50). Under these circumstances, bacterial translocation from the gut without proper control by mucosal immunity has the potential to have a systemic effect (51). Despite observing increased gut permeability in this 7-day DSS model, we found minimal changes in serum cytokine levels in both WT and *J*α*18^−/−^* mice. This absence of systemic inflammation is highly suggestive that systemic bacterial dissemination had not occurred (52). As further evidence of this, we examined the behavior of iNKT cells in the liver. Any bacteria entering the host from the colon are filtered through the liver (37), where they can be captured by resident liver immune cells (28), and activate hepatic iNKT cells (28, 53). In support of our results in unchanged systemic cytokine levels, we did not observe altered iNKT cell behavior in the liver post-DSS. Taken together, these findings indicate that in this model of DSS-induced colitis, inflammation is restricted to the colon.

In addition, we observed more colonic neutrophils in mice deficient of iNKT cells post-DSS, but whether this finding is attributed only by the local inflammatory response or by the altered gut microbiota is unknown. Therefore, we conducted two experiments, whereby we depleted iNKT cells with NKT14 or inhibited iNKT activation with α-CD1d, within the same strain of animals. Remarkably, depletion of iNKT cells resulted in increased numbers of adherent neutrophils in the colonic microvasculature at day 3 and day 5 of our DSS model. To our knowledge, this is the first study to assess the temporal profile of neutrophil recruitment in DSS-induced inflammation. Moreover, we also found increased neutrophil numbers at day 7 of our DSS model following the blockade of CD1d-presentation. These findings indicate that iNKT cells have an active role in suppressing neutrophil recruitment to the colon *via* CD1d and provide evidence that these effects are independent of the bacterial composition within the gut microbiota.

Another notable finding was the intense neutrophil infiltration within 3 days of DSS administration, while clinical symptoms were not observed until day 5. This uncoupling between infiltrating neutrophils and clinical symptoms echoes the unexpected observations of increased neutrophil numbers associated with decreased pathology in post-DSS *J*α*18^−/−^* mice. The inverse relationship between colonic neutrophils and disease pathology suggests that neutrophils have more complex roles than previously hypothesized. Although prior studies have suggested against pathological roles of neutrophils (3, 5), recent evidence indicates that similar to macrophages, neutrophils can adopt pro-inflammatory or anti-inflammatory phenotypes (41). The increase in *Ym1* expression in isolated colonic neutrophils in post-DSS *J*α*18^−/−^* mice suggests polarization toward an anti-inflammatory phenotype that may confer tissue protection (43). While iNOS (*Nos2*) is not classically considered as a marker of anti-inflammation, it has been shown to exert suppressive effects on T cells (42). Furthermore, in a colonic environment damaged by DSS, it is highly probable that recruited neutrophils have important bactericidal functions regulated by the expression of *Nos2* (54). Indeed, previous studies have shown neutrophils can alleviate colonic inflammation induced by *Clostridium difficile* infection (55, 56). Therefore, our findings suggest that the colonic neutrophils of post-DSS *J*α*18^−/−^* mice can mediate anti-inflammatory effects in two ways, directly *via* genes expressed under the anti-inflammatory phenotype and indirectly by elimination of invading bacteria.

Due to the differences between the neutrophil populations in WT and *J*α*18^−/−^* mice, we hypothesized that depletion of neutrophils will have opposing effects in the two strains of mice post-DSS. Indeed, our results show reduced shortening of colon lengths in WT mice depleted of neutrophils, and more severe disease in *J*α*18^−/−^* mice depleted of neutrophils. More importantly, after neutrophil depletion, the resistance to DSS-induced colitis in mice lacking iNKT cells is nullified. This indicates that neutrophils have important and non-redundant anti-colitogenic effects in a microenvironment that lacks iNKT cells. Interestingly, our results showed that IL-1β, CXCL1, and CXCL2, but not TGF-β, are produced by colon infiltrating neutrophils and are likely to perform important autocrine functions on neutrophils to further their recruitment. Numerous previous studies have identified Tregs as important regulators of inflammation in the gastrointestinal tract (57). However, in this study, our results suggested that this was not the case in this acute phase of the disease model. In the absence of iNKT cells and neutrophils, we observed an increase in the number of Tregs in the colon, although this was not associated with a reduction in pathology. Taken together, these observations suggest that in the acute phase of colonic injury induced by DSS, neutrophils confer greater anti-inflammatory protection than Tregs.

**Figure 7:**
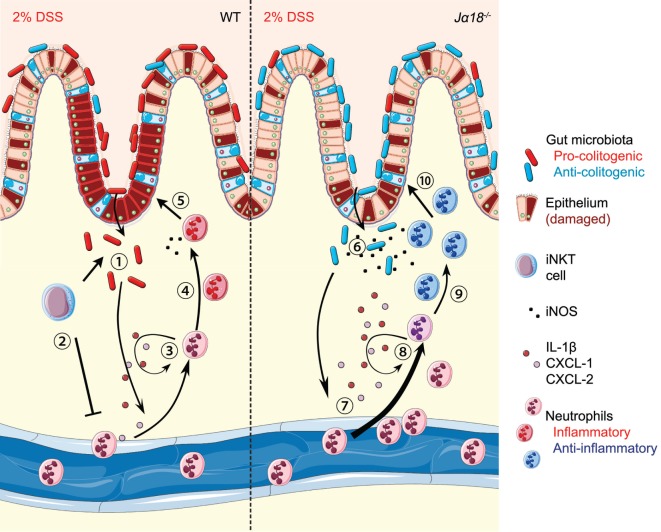
Schematic diagram showing the invariant natural killer T (iNKT) cell–microbiota–neutrophil axis in regulation of dextran sodium sulfate (DSS)-induced intestinal inflammation. (1) Continual administration of 2% DSS in wild-type (WT) mice results in epithelial damage and allows translocation of inherently pro-colitogenic gut bacteria. This results in local colonic inflammation, production of IL-1β, CXCL1, and CXCL2, and the recruitment of immune cells, including neutrophils. (2) Antigen presentation of the translocated bacteria to iNKT cells acts to suppress exacerbated recruitment of neutrophils. (3) Once recruited, neutrophils produce IL-1β, CXCL1, and CXCL2, which function in an autocrine manner for further recruitment. (4) In the highly inflammatory environment, neutrophils polarize toward a pro-inflammatory phenotype. (5) This inflammatory phenotype, in combination with low production of bactericidal iNOS, is detrimental and results in increased intestinal inflammation. (6) In *J*α*18^−/−^* mice, there is translocation of anti-colitogenic gut bacteria. (7) Without iNKT cells, there is earlier and more pronounced recruitment of neutrophils to the colon. (8) The recruitment is likely sustained due to the autocrine functions of IL-1β, CXCL1, and CXCL2. (9) The presence of anti-colitogenic bacteria supports neutrophils to function toward an anti-inflammatory phenotype. (10) Increased production of iNOS by neutrophils enables dual bactericidal and immune-modulatory functions, thereby resulting in lower disease pathology. (Stock images sourced from Servier Medical Art; Creative Commons)

Collectively, our results demonstrate important functions of the iNKT cell–microbiota and iNKT cell–neutrophil interactions in regulating colonic inflammation (Figure 7). In DSS-treated WT mice, pro-colitogenic bacteria potentiate a pro-inflammatory microenvironment that is restricted to the colon. In the event of epithelial damage and/or bacterial translocation, iNKT cells are activated in a CD1d-dependent manner to suppress the excessive recruitment of potentially pro-inflammatory neutrophils. In *J*α*18^−/−^* mice, the absence of iNKT cells increases the recruitment of neutrophils, likely due to increased expressions of IL-1β, CXCL1, and CXCL2, which acts in an autocrine fashion to potentiate neutrophil recruitment. However, the lack of iNKT cells also shapes the gut microbiota toward an anti-colitogenic phenotype, directing neutrophils to an anti-inflammatory phenotype and protection of disease. Therefore, we demonstrated overarching roles of the gut microbiota in regulating the colonic microenvironment, and synergistic roles with iNKT cells in polarization of neutrophil function. Our findings demonstrate that the lack of iNKT cells alters the gut microbiota to create a microenvironment that is less inflammatory, while supporting the recruitment of anti-inflammatory neutrophils, which jointly protects the host from DSS-induced inflammation. Future research that provides further insight into the iNKT cell–microbiota–neutrophil axis has the potential to identify novel therapeutic avenues to reduce the impact of colitis.

## Data Deposition Statement

All of the sequencing data are submitted to the Metagenomics Analysis Server (MG-RAST) under library mgl621715, titled “Invariant natural killer T cells regulate neutrophil recruitment and function in colitis.”

## Ethics Statement

All experimental procedures were performed in accordance with protocols approved by the Monash Medical Centre B Animal Ethics Committee (MMCB/2015/15).

## Author Contributions

CW, MH, and SS conceived and planned the experiments. SS, with the help of KPK, SWW, and CW, carried out the experiments and analysis. KPK performed gut immunofluorescence experiments and analysis. For gut microbiota analysis, SS contributed to sample preparation, RM and TTHV performed sequencing, and DS performed analysis and interpretation of the results. SS and CW wrote the manuscript with input from all authors.

## Conflict of Interest Statement

The authors declare that the research was conducted in the absence of any commercial or financial relationships that could be construed as a potential conflict of interest.
