# Supplementary material for: Invariant Natural Killer T Cells Shape the Gut Microbiota and Regulate Neutrophil Recruitment and Function During Intestinal Inflammation
Source: Front Immunol. 2018 May 7;9:999. doi: 10.3389/fimmu.2018.00999 (PMC5949322; doi:10.3389/fimmu.2018.00999)
Supplement: Supplementary file 3 [file data_sheet_1.PDF]

Invariant natural killer T cells shape the gut microbiota and regulate neutrophil recruitment and function during intestinal inflammation

Sj Shen[1], Kathryn Prame Kumar[1], Dragana Stanley[2], Robert J. Moore[3, 4], Thi Thu Hao Van[4], Shu Wen Wen[1], Michael J. Hickey[1] and Connie H. Y. Wong[1, *]

[1] Centre for Inflammatory Diseases, Monash University Department of Medicine, Monash Medical Centre, Clayton, Victoria, Australia;

[2] School of Health Medical and Applied Sciences, Central Queensland University, Australia;

[3] Infection and Immunity Program, Monash Biomedicine Discovery Institute and Department of Microbiology, Monash University, Australia;

[4] School of Science, RMIT University, Australia;

*Address for correspondence: Connie H. Y. Wong, PhD., Centre for Inflammatory Diseases, Department of Medicine, School of Clinical Sciences at Monash Health, Monash Medical Centre, Monash University, Clayton, VIC 3168 Australia; Email: connie.wong@monash.edu

Running title: iNKT cells regulate neutrophil recruitment and function

Key words: neutrophils, colitis, inflammation, mucosal immunology, innate immunity

Supplemental materials

Supplemental tables

Supplemental Table 1. Primer sets used for gene expression analysis by qPCR

Primer		Sequence
18S	F	CTT AGA GGG ACA AGT GGC G
	R	ACG CTG AGC CAG TCA GTG TA
Chil3 (Ym-1)	F	AAG CTC TCC AGA AGC AAT CCT G
	R	GAA GAA TTG CCA GAC CTG TGA C
Cxcl1	F	CCG AAG TCA TAG CCA CAC TCA A
	R	GCA GTC TGT CTT CTT TCT CCG TTA C
Cxcl2	F	CCA ACC ACC AGG CTA CAG G
	R	GCG TCA CAC TCA AGC TCT G
Il1b	F	ATG GCA ACT GTT CCT GAA CTC AAC T
	R	CAG GAC AGG TAT AGA TTC TTT CCT TT
Il18	F	CAG GCC TGA CAT CTT CTG CAA
	R	CTG ACA TGG CAG CCA TTG T
Mrc1 (CD206)	F	AGG CTG ATT ACG AGC AGT GG
	R	CGC CCA GTA TCC ATC CTT GC
Nos2 (iNOS)	F	GCT TGG GTC TTG TTC ACT CC
	R	GCA AGT GAA ATC CGA TGT GGC
Tgfb	F	GAG CCA GAA CGA GAA GTA CCG
	R	CCT CAA GAC GAG CAA TTT CAT CA
Tnfa	F	ATG AGC ACA GAA AGC ATG ATC CGC
	R	CCA AAG TAG ACC TGC CCG GAC TC

Supplemental figures

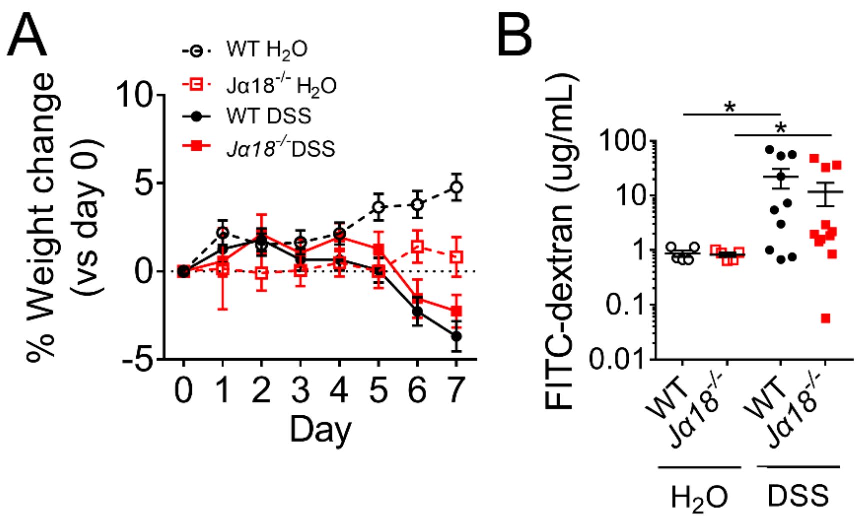

Supplemental Figure 1. (A) Changes in weight of WT and *Jα18*$^{-/-}$ mice provided with normal drinking water (H_2O groups) or treated with 2% DSS (DSS groups) were normalised to day 0. (B) Gut permeability of WT and *Jα18*$^{-/-}$ mice. FITC-dextran was administered by oral gavage, and its levels in the serum was analysed in WT and *Jα18*$^{-/-}$ mice administered with H_2O or 2% DSS. n≥5. Data represent mean ± SEM. Student's *t* test was performed. These data were obtained from at least 2 independent experiments.

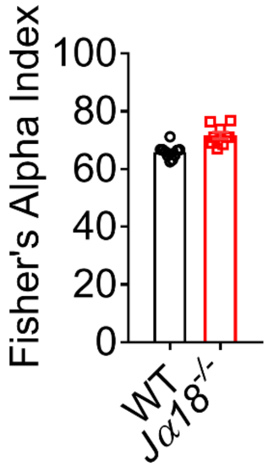

Supplemental Figure 2. α-diversity of the gut microbiota in WT and *Jα18*$^{-/-}$ mice. n≥9. Data represent mean ± SEM. These data were obtained from one experiment (2 independent cages/group).

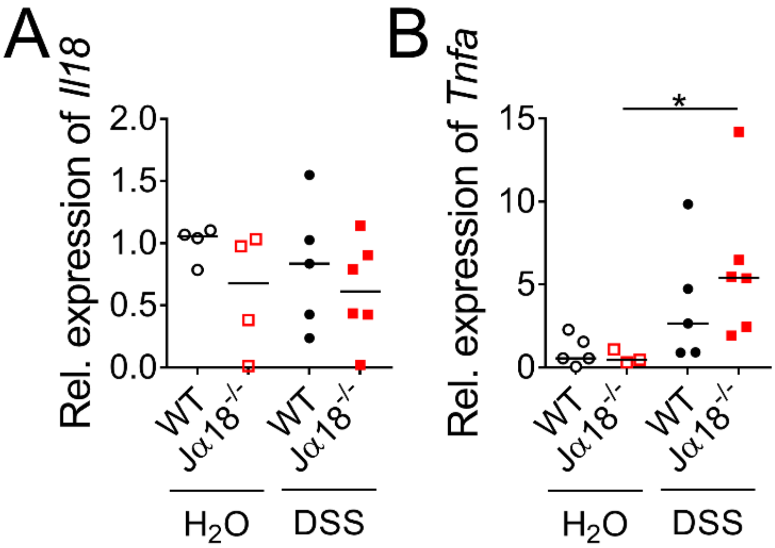

Supplemental Figure 3. Local effects of DSS administration in WT and *Jα18*$^{-/-}$ mice. (A, B) Expressions of genes in the colon were quantified by qPCR and expressed relative to WT H_2O controls. n≥4. Lines represent medians, and Mann-Whitney U-test was performed. Significance is represented by $^*p<0.05$. These data were obtained from at least 2 independent experiments.

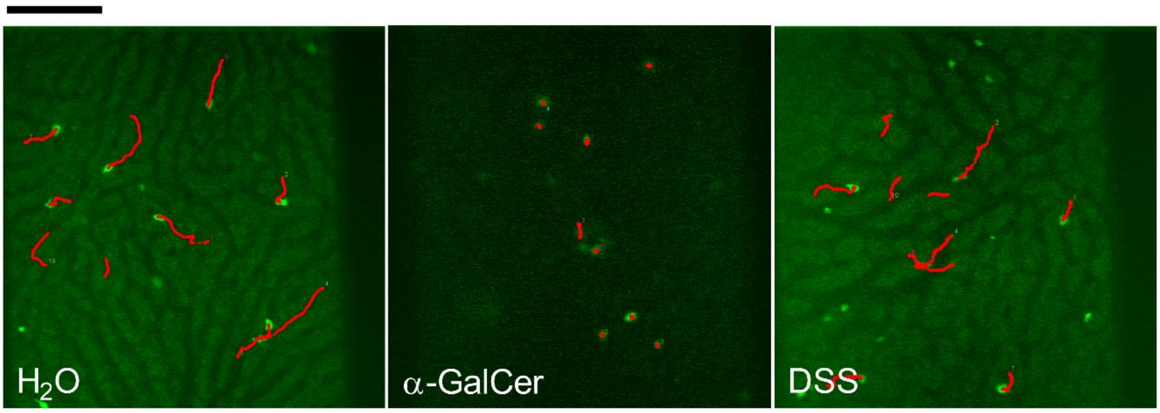

Supplemental Figure 4. Representative images showing tracking of iNKT cells in the liver of *Cxcr6*$^{gfp/+}$mice. The use of MTrackJ plugin enabled the tracking of displacement of iNKT cells in the liver of *Cxcr6*$^{gfp/+}$mice on H_2O control, injected with α-GalCer i.p., or after 7 days of 2% DSS. Bright green cells are iNKT cells; red lines represent paths. Scale bar represents 100 μm.

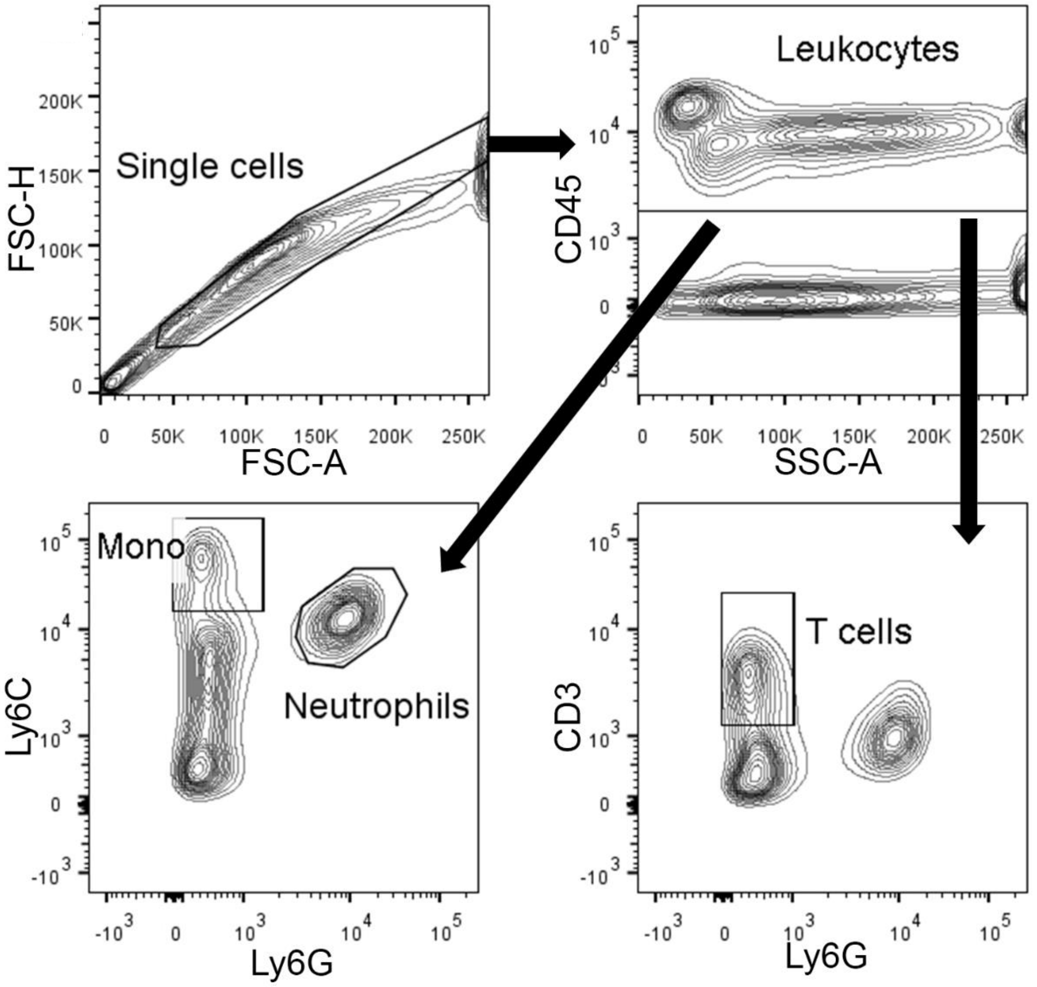

Supplemental Figure 5. Gating strategy used to examine leukocyte populations following DSS-induced colitis. Mono: Ly6C^{hi} monocytes

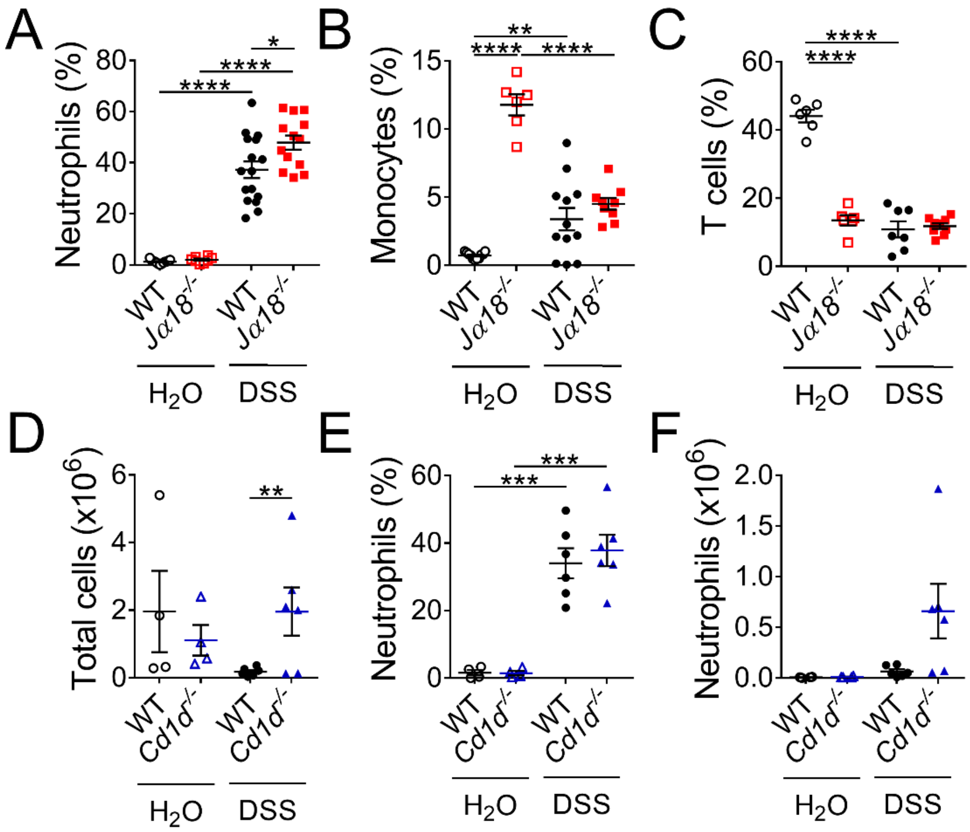

Supplemental Figure 6. Changes in leukocyte populations following DSS-induced colitis in WT, *Jα18*$^{-/-}$ and *Cd1d*$^{-/-}$ mice. (A-C) At day 7, leukocytes were isolated from colon of WT and *Jα18*$^{-/-}$ mice and stained with fluorochrome-conjugated antibodies against CD45, CD3, Ly6C, and Ly6G. The proportion (of CD45^{+} cells) of (A) Ly6G^{+} neutrophils, (B) Ly6C^{+}Ly6G^{-} monocytes, and (C) CD3^{+} T cells were analysed by flow cytometry. (D) At day 7, total leukocyte numbers from colon of WT and *Cd1d*$^{-/-}$ mice were counted. (E) The percentage (of CD45^{+} cells) and (F) number of Ly6G^{+} neutrophils were analysed by flow cytometry. n≥2. Data represent mean ± SEM. Student's *t* test was performed. Significance is represented by *p<0.05, **p<0.01, ***p<0.001 and ****p<0.0001. These data were obtained from at least 2 independent experiments.

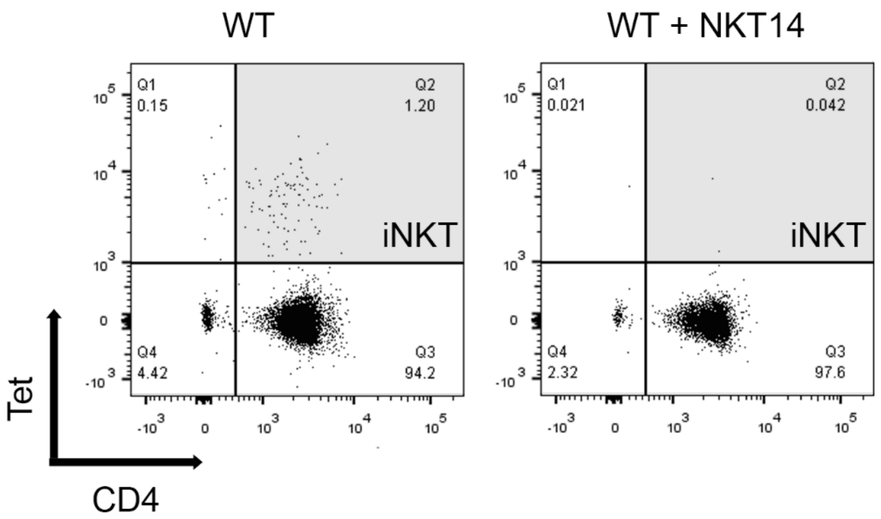

Supplemental Figure 7. Representative plot showing depletion of CD4$^+$ Tetramer (Tet)$^+$ iNKT cells in the spleen of WT mice 7 days after NKT14 injection. The population is gated from CD45$^+$ CD3$^+$ T cells

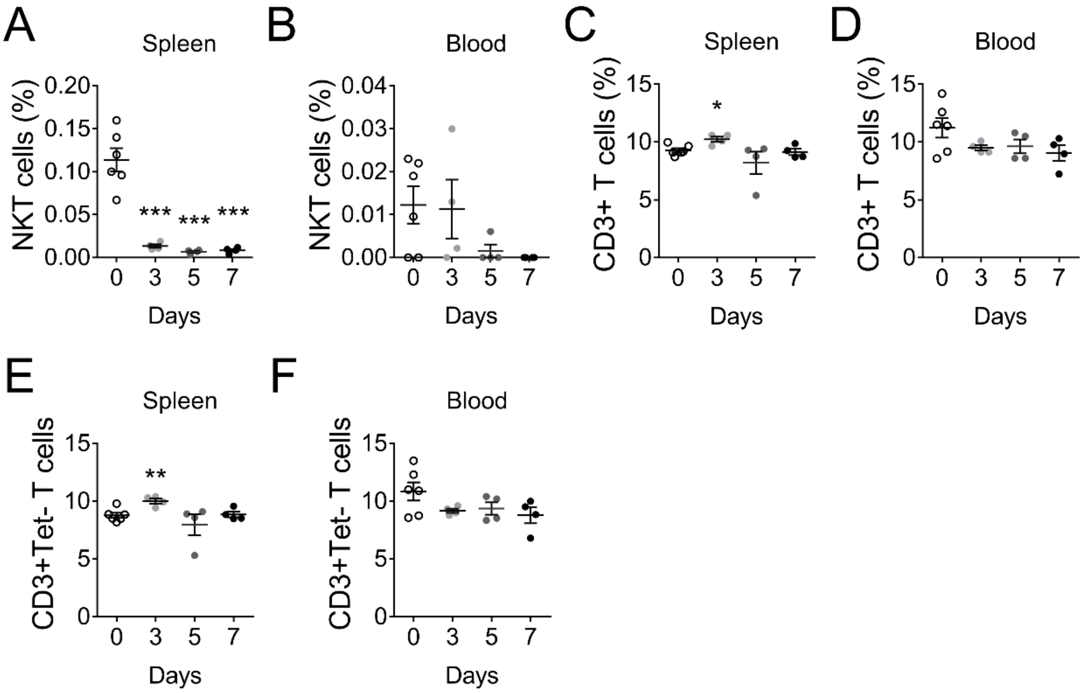

Supplemental Figure 8. Effect of NKT14 treatment in WT mice on T cell populations over 7 days. (A) $CD45^+$ $CD3^+$ $CD4^+$ Tetramer $(Tet)^+$ NKT cells in the spleen and (B) blood, (C) all T cells in the spleen and (D) blood, and (E) T cells that are Tet- in the spleen and (F) blood were assessed at days 0, 3, 5, and 7 by flow cytometry. n≥4. Data represent mean ± SEM. Student's *t* test was performed. Significance (vs day 0) is represented by *p<0.05, **p<0.01, ***p<0.001. These data were obtained from at least 2 independent experiments.

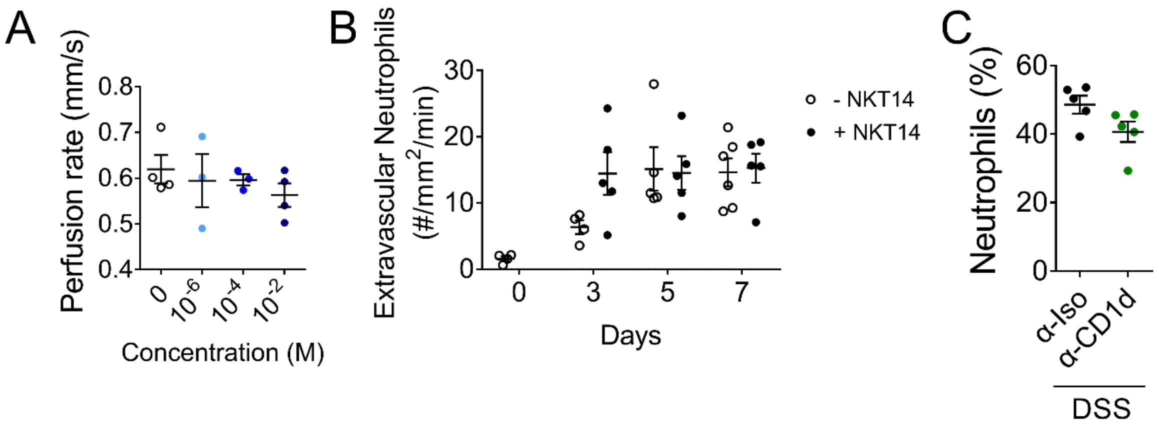

Supplemental Figure 9. Use of atropine superfusion in intravital imaging of colonic neutrophils. (A) The colon of WT mice was superfused with increasing concentrations of atropine, and blood perfusion rate was measured by fluorescent beads using spinning disk confocal microscopy. (B) Extravascular neutrophils were quantified in *LysM*^{eGFP} mice administered with or without NKT14. (C) The percentage of neutrophils in DSS-treated WT mice with or without CD1d-blocking antibody was assessed by flow cytometry. n≥3. Data represent mean ± SEM. Student's *t* test was performed relative to (A) 0 M, (B) - NKT14 group, or (C) isotype control (α-Iso). No statistical significances were observed. These data were obtained from at least 2 independent experiments.

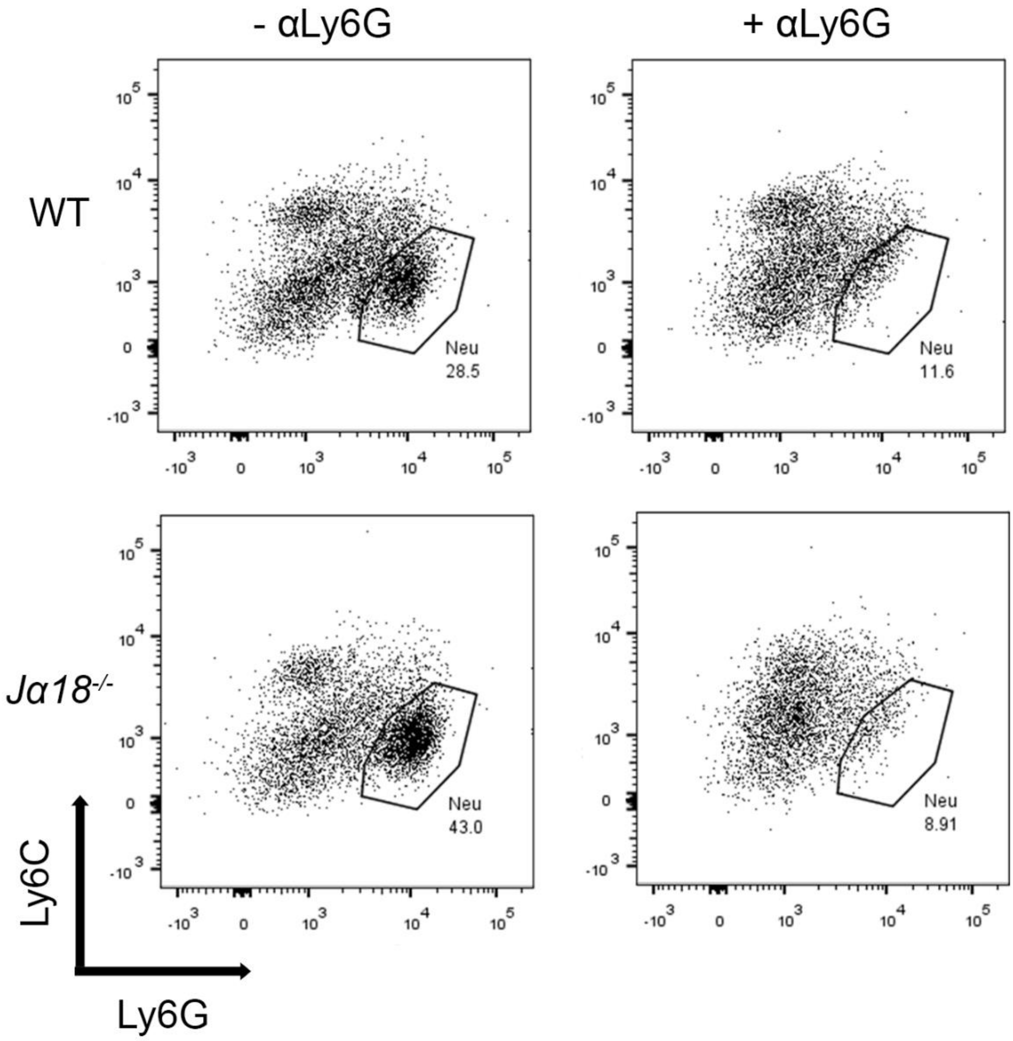

Supplemental Figure 10. Representative plot showing depletion of Ly6C+ Ly6G+ neutrophils in the spleen of WT and *Jα18*$^{-/-}$ mice following injections of αLy6G and 7 days of DSS treatment.

Supplemental Movie 1. Multiphoton time-lapse video of *LysM*^{eGFP} mouse colon microvasculature at baseline. There are minimal neutrophil-endothelial interactions, and few cells outside of the vasculature and in the tissue. The time-lapse covers a period of 3 mins, with approximately 15 s elapsed per second of movie.

Supplemental Movie 2. Multiphoton time-lapse video of *LysM*^{eGFP} mouse colon microvasculature at day 7 post-DSS administration. Neutrophils are interacting with the endothelium, resulting in increased rolling and adhesion. Additionally, there are also increased extravascular neutrophils. The time-lapse covers a period of 3 mins, with approximately 15 s elapsed per second of movie.
